# Association between fetal eye movement density and developmental problems at age 3 years

**DOI:** 10.1038/s41598-026-35780-3

**Published:** 2026-01-16

**Authors:** Yukiyo Shimada, Seiichi Morokuma, Kazushige Nakahara, Akiko Okuno, Kiyoko Kato

**Affiliations:** 1 Department of Obstetrics and Gynecology, Graduate School of Medical Sciences, Kyushu University, Fukuoka, Japan; 2 Department of Health Sciences, Graduate School of Medical Sciences, Kyushu University, Fukuoka, Japan; 3 Center for Baby Science, Doshisha University, Kyoto, Japan

**Keywords:** Eye movement density, Fetal neurodevelopment, Rapid eye movement sleep, Language development, Autism-related traits, Sleep problem, Biomarkers, Diseases, Medical research, Neurology, Neuroscience

## Abstract

In this longitudinal study of 77 pregnant women, we examined how eye movement density (EMD) measured at 34 to 36 weeks’ gestation related to child development at age 3 years, using two developmental assessment tools. Fetal eye movements were recorded for 60 min. Data from 41 children with complete follow-up at age 3 years were analyzed. EMD was calculated as the number of eye movements per minute during periods with eye movement activity, which reflects the active behavioral state considered a precursor to rapid eye movement (REM) sleep. At 3 years, development was assessed using the Kinder Infant Development Scale and autism-related traits using the Social Responsiveness Scale-2. Sleep was evaluated with caregiver-completed questionnaires and sleep logs at 6 months, 1 year, and 3 years. Higher fetal EMD was associated with greater receptive and expressive language development, whereas lower EMD was linked to higher restricted and repetitive behavior (RRB) scores and an increased likelihood of clinically significant RRB. Moreover, lower EMD predicted later bedtimes at age 1 year. These findings suggest that fetal EMD reflects early maturation of neural circuits and may serve as a noninvasive early biomarker for identifying children at risk of developmental and behavioral difficulties.

**Supplementary Information:**

The online version contains supplementary material available at 10.1038/s41598-026-35780-3.

## Introduction

Fetal eye movements (EMs) can be detected by ultrasonography at 14 weeks of gestation^1^.By approximately 23 weeks, these movements become more organized, and rapid eye movements (REMs), a characteristic feature of active sleep, can be detected^2^. From 28 to 37 weeks, eye movement density (EMD)—the frequency of eye movements per unit time—demonstrates a developmental increase, reflecting the maturation of brainstem mechanisms that generate active sleep–related activity^3^. In parallel with this trajectory, the differentiation between active and quiet sleep states, which represent the precursors of mature REM and non-REM (NREM) sleep states^4^, emerges around 30 weeks, marking the establishment of active–quiet cycling in utero^5^. Because mature REM and NREM stages cannot be reliably distinguished until several months after birth, early sleep—including fetal sleep—is interpreted behaviorally. Active sleep, the precursor of REM, is characterized by irregular respiration and brisk eye movements^4^. Therefore, fetal eye movements are considered behavioral markers of REM-related precursor activity. These findings indicate that sleep-related neural activity begins before birth.

REM sleep plays a crucial role in brain plasticity during development^6^. As EMD reflects REM activity, it may serve as an early indicator of neurodevelopmental disorders. This is supported by studies on autism spectrum disorder (ASD), in which infants often exhibit early sleep disturbances with lower REM activity than typically developing children^7,8^. A previous study reported a relationship between REM activity in preterm neonates and neurodevelopmental outcomes at 6 months’ corrected age^9^. Therefore, EMD may be a predictor of neurodevelopmental disorders^10^. Moreover, these findings suggest that sleep abnormalities occur during the fetal period. Fetal EMD has been associated with sleep and developmental outcomes at 1.5 years^11^, demonstrating the importance of early REM activity as a potential predictor of neurodevelopmental outcomes. Fetal eye movements encompass both slow and rapid movements, and not all eye movements necessarily represent the rapid eye movements characteristic of mature REM sleep^12^. Nevertheless, the overall density of eye movement activity during active states has been demonstrated to reflect neurodevelopmental maturation^3^.

Developmental assessments at 3 years of age are considered important because core functions such as language and behavioral regulation are sufficiently differentiated by this stage^13^. This age also marks a period when behavioral patterns become more stable and reliably measurable, allowing for meaningful interpretations in clinical and research settings^14^. By 3 years of age, developmental assessments covering a wider range of domains can be conducted, including language, behavioral regulation, and social communication, which are insufficiently developed for reliable measurement at earlier age. The Kinder Infant Development Scale (KIDS) is a screening questionnaire designed to reflect the typical Japanese lifestyle and daily behaviors^15,16^; therefore, it was considered suitable for evaluating developmental domains in this cohort.

In addition, the Social Responsiveness Scale-2 (SRS-2), a screening questionnaire for autism spectrum disorder, can be applied from 2.5 years of age^17^.

However, no studies have examined the long-term impact of fetal EMD on neurodevelopment at 3 years of age. To clarify the relationship between fetal neurological development and developmental outcomes at 3 years of age, we aimed to examine the relationship between EMD measured at 34 to 36 weeks of gestation (as described in a previous study)^11^ and caregiver-reported scores on two developmental assessment tools at age 3 years: the KIDS and SRS-2. In addition, sleep status was assessed at 6 months, 1 year, and 3 years to investigate its longitudinal pattern in relation to fetal EMD.

## Results

**Fig. 1:**
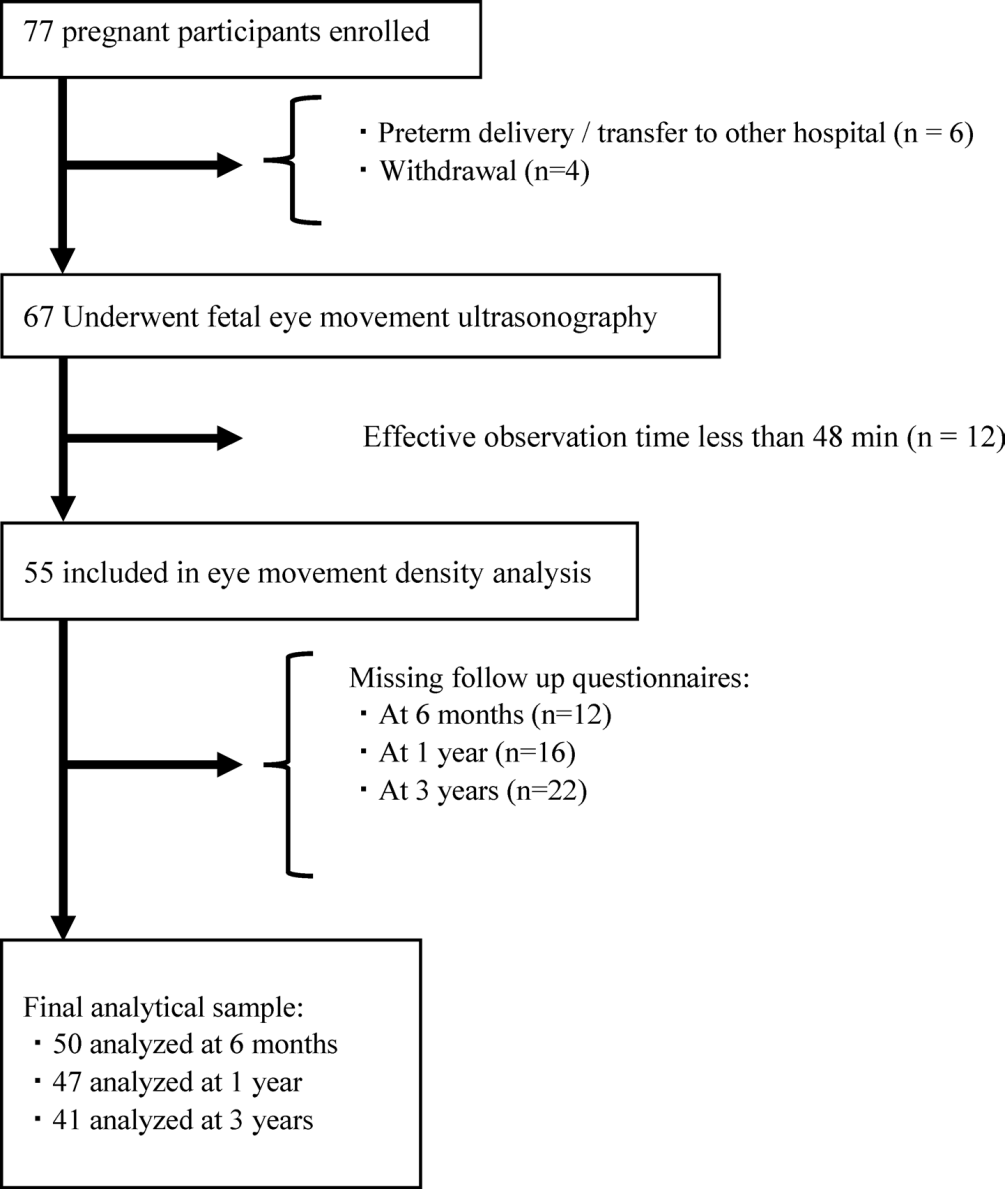
Participant Flow.

A total of 77 pregnant women were recruited for this study. Fetal EM was successfully examined in 67 participants; 10 were excluded because of preterm delivery, transfer to another hospital (*n* = 6), or withdrawal from the study (*n* = 4). Owing to insufficient ultrasound observation time (< 48 min; *n* = 12) or missing questionnaire responses at follow-up (6 months, *n* = 12; 1 year, *n* = 16; 3 years, *n* = 22), several participants were excluded from the developmental analysis, with some overlapping across time points. Consequently, among participants with valid fetal EMD measurements, outcome data (that is, follow-up questionnaire responses) were available for 50 at 6 months, 47 at 1 year, and 41 at 3 years, with the latter constituting the final analytical sample for developmental assessment (Fig. 1). For the 3-year sleep assessment, sleep logs were considered acceptable if entries were recorded on at least 10 out of 14 consecutive days; based on this criterion, sleep data from 37 participants were included in the analysis. The mean gestational age at the time of fetal EM assessment was 34.7 weeks (range: 34–36 weeks’ gestation), and the average EMD was 10.3 movements per minute (standard deviation [SD] = 3.5; range: 4.4–16.5). The mean chronological age at the time of response to the 3-year questionnaire was 36.2 months ([SD] = 0.7, range: 35‒39) (Table 1). Supplementary Table S1 displays the comparison of demographic characteristics between the follow-up and attrition groups, indicating no significant differences.

**Table 1:** Baseline characteristics and outcomes of the study population (41 cases).

				Mean or n	SD (%)	Range
Maternal characteristics						
Maternal age at delivery(years)				36.3	5.7	21‒46
Parity						
		0 (n)		26	(63.4%)	
		≧ 1 (n)		15	(36.6%)	
Ultrasonographic measurements						
Gestational age at examination (weeks)				35.0	0.6	34.0‒36.4
Effective observation time (min)				57.0	2.8	50.7‒63.4
EM period (min)				34.7	8.5	17‒52
EM density (n/min)				10.3	3.5	4.4‒16.5
Birth information						
Gestational age at birth (weeks)				39.0		36.4‒41.4
Type of delivery						
		Vaginal (n)		23	(56.1%)	
		Caesarean (n)		18	(43.9%)	
Birth Weight (g)				2945.3	357.9	1982‒3656
Sex						
		Male (n)		16	(39.0%)	
		Female (n)		25	(61.0%)	
Apgar score at 5 min				9.0	0.4	8‒10
pH of the umbilical artery				7.298	0.055	7.194‒7.466
Age at time of response to this questionnaire (month)						
			3 years	36.2	0.7	35‒39

SD, standard deviations; EM, eye movement.

### Association between fetal EMD and KIDS

Table 2 presents the results of the linear regression analyses examining the association between fetal EMD and developmental age across the KIDS domains. Among them, receptive (mean: 42.9 months; range: 26–46 months) and expressive (mean: 41.9 months; range: 27–48 months) languages were significantly associated with fetal EMD. In the univariate model, the β coefficients were 0.36 (95% CI [confidence interval]: 0.08‒0.91, *p* = 0.021) for receptive language and 0.38 (95% CI: 0.14‒1.17, *p* = 0.014) for expressive language. These associations remained significant in the multivariate model adjusted for maternal age at delivery and birth weight (receptive: β = 0.36, 95% CI: 0.07‒0.91, *p* = 0.024; expressive: β = 0.38, 95% CI: 0.13‒1.19, *p* = 0.017). No significant associations were observed between fetal EMD and other developmental domains. These findings suggest a significant positive association between fetal EMD and language development.

**Table 2:** Association between fetal EMD and KIDS developmental age (months).

		Number of responses	Mean*	Range	Univariate model			Multivariate model**		
			(month)		β	95%CI	*p* Value	β	95%CI	*p* Value
Physical-motor		41	38.1	33‒45	−0.01	−0.33 to 0.24	0.746	−0.06	−0.35 to 0.24	0.706
Manipulation		41	40.8	25‒47	0.21	−0.20 to 0.96	0.196	0.20	−0.22 to 0.97	0.213
Receptive language		41	42.9	26‒46	0.36	0.08 to 0.91	0.021	0.36	0.07 to 0.91	0.024
Expressive language		41	41.9	27‒48	0.38	0.14 to 1.17	0.014	0.38	0.13 to 1.19	0.017
Linguistic concept		41	43.7	33‒47	0.23	−0.09 to 0.58	0.146	0.23	−0.01 to 0.59	0.158
Social relationships with children		41	44.6	35‒48	0.22	−0.11 to 0.63	0.160	0.22	−0.12 to 0.64	0.177
Social relationships with adults		41	40.6	30‒45	0.24	−0.09 to 0.62	0.138	0.24	−0.10 to 0.63	0.148

EMD, eye movement density; KIDS, The Kinder Infant Development Scale; β, partial regression coefficient; 95% CI, 95% confidence interval.

Univariate and multivariate linear regression analyses were performed to examine the association between fetal EMD and KIDS developmental age (months).

* Mean developmental age in months.

**Adjusted for maternal age at delivery and birth weight.

### Association between fetal EMD and SRS-2

Table 3 summarizes the results of the linear regression analyses examining the association between fetal EMD and SRS-2 T-scores. Among the subscales, repetitive behavior (RRB) scores showed a significant association with fetal EMD (mean T-score: 49.8; range: 37–90). In the univariate model, a significant negative association was found (β = -0.33, 95% CI: -1.83 to -0.06, *p* = 0.037), which remained significant in the multivariate model adjusted for maternal age at delivery and birth weight (β = -0.33, 95% CI: -1.83 to -0.07, *p* = 0.035). Table 4 presents the results of the logistic regression analysis assessing the likelihood of elevated scores (T-score ≥ 60) across the SRS-2 subscales. Among these, a significant association was observed only for RRB, with lower fetal EMD predicting increased odds of elevated RRB (Odds ratio [OR] = 0.48, 95% CI: 0.17 to 0.83, *p* = 0.003). Notably, RRB was the only subscale that showed significant associations with fetal EMD across all three models: univariate, multivariate, and logistic regression. No significant associations were observed between fetal EMD and the other subscales in either the linear or logistic regression models. These findings suggest that lower fetal EMD is significantly associated with higher RRB scores during early childhood.

**Table 3:** Association between fetal EMD and SRS-2 T-score.

	Number of responses*	Mean	Range	Univariate model			Multivariate model**		
				β	95%CI	*p* Value	β	95%CI	*p* Value
Total score	34	49.4	32‒88	−0.17	−1.71 to 0.61	0.342	−0.19	−1.78 to 0.51	0.266
Social awareness	39	48.8	26‒72	−0.02	−1.20 to 1.05	0.898	−0.03	−1.23 to 1.05	0.875
Social cognition	36	49.8	33‒85	−0.19	−1.65 to 0.46	0.261	−0.20	−1.65 to 0.43	0.238
Social communication	36	48.2	33‒72	−0.10	−1.12 to 0.61	0.552	−0.11	−1.13 to 0.58	0.511
Social motivation	40	50.8	32‒70	−0.03	−1.07 to 0.86	0.832	−0.03	−1.08 to 0.88	0.832
Restricted interests and repetitive behavior	40	49.8	37‒90	−0.33	−1.83 to −0.06	0.037	−0.33	−1.83 to −0.07	0.035
Social communication and interaction	34	49.2	30‒80	−0.14	−0.16 to 0.70	0.437	−0.16	−1.63 to 0.60	0.350

EMD, eye movement density; SRS-2, the Social Responsiveness Scale-2; β, partial regression coefficient; 95% CI, 95% confidence interval.

Univariate and multivariate linear regression analyses were performed to examine the association between fetal EMD and SRS-2 T-scores.

*The number of responses varied across the SRS-2 domains because incomplete responses were excluded from the analysis, leading to different sample sizes for each domain.

**Adjusted for maternal age at delivery and birth weight.

**Table 4:** Association between fetal EMD and SRS-2 abnormality.

	Number of responses*	Number of outcomes**	%	Univariate model		
				OR	95%CI	*p* Value
Total score	34	5	14.7	0.83	0.57 to 1.13	0.248
Social awareness	39	7	17.9	0.98	0.76 to 1.25	0.870
Social cognition	36	3	8.3	1.00	0.67 to 1.45	0.999
Social communication	36	3	8.3	0.65	0.26 to 1.05	0.085
Social motivation	40	7	17.5	0.89	0.67 to 1.13	0.342
Restricted interests and repetitive behavior	40	5	12.5	0.48	0.17 to 0.83	0.003
Social communication and interaction	34	5	14.7	0.83	0.57 to 1.13	0.248

EMD, eye movement density; SRS-2, the Social Responsiveness Scale-2; OR, odds ratio (per unit odds ratio); 95% CI, 95% confidence interval.

Univariate logistic regression was performed with a cutoff of 60, categorizing scores ≥ 60 as abnormal and < 60 as normal.

*The number of responses varied across the SRS-2 domains because incomplete responses were excluded from the analysis, leading to different sample sizes for each domain.

** Number of cases classified as positive (coded as 1) based on the cutoff.

### Association between fetal EMD and sleep problems at 6 months, 1 year, and 3 years

We examined the association between fetal EMD and sleep problems at the ages of 6 months, 1 year, and 3 years using univariate logistic regression (Table 5). At 1 year, a lower EMD was significantly associated with a higher likelihood of bedtime after 22:00 (*p* = 0.014). A similar trend was observed at 6 months (*p* = 0.078) and 3 years (*p* = 0.108), although the difference did not reach statistical significance. No significant associations were found between EMD and either nighttime sleep duration (≤ 9 h) or night awakenings at any age.

**Table 5:** Association between fetal EMD and sleep problems at 6 months, 1 year, and 3 years.

		Number of outcomes**		Univariate analysis		
	Number of responses*	*n*	%	OR	95% CI	*p* value
6 months						
Bedtime after 22:00	50	12	24.0	0.83	0.65–1.01	0.078
Sleep for ≦ 9 h during the night (20:00‒8:00)	50	27	54.0	0.92	0.78–1.08	0.304
Night awaking	49	4	8.2	0.94	0.67–1.26	0.697
12 months						
Bedtime after 22:00	47	10	21.3	0.68	0.47–0.89	0.014
Sleep for ≦ 9 h during the night (20:00‒8:00)	47	23	48.9	0.93	0.78–1.11	0.428
Night awaking	47	5	10.6	1.18	0.89–1.64	0.261
3 years						
Bedtime after 22:00	37	10	27.0	0.81	0.61–1.03	0.108
Sleep for ≦ 9 h during the night (20:00‒8:00)	37	7	18.9	1.01	0.78–1.30	0.952
Night awaking	37	7	18.9	1.08	0.84–1.41	0.542

EMD, eye movement density; OR, odds ratio (per unit odds ratio); 95% CI, 95% confidence interval.

Univariate logistic regression was performed to examine the association between fetal EMD and each sleep indicator.

* Sample size differs between domains because of missing answers.

** Number of cases classified as positive (coded as 1) based on the cutoff.

## Discussion

We investigated the association between fetal EMD and neurodevelopmental outcomes at 3 years of age. Our findings suggest that lower fetal EMD is associated with higher RRB scores, whereas higher fetal EMD is linked to more favorable development in receptive and expressive language abilities. In addition, lower fetal EMD was associated with later sleep onset time at 1 year of age.

These findings are consistent with those of previous studies showing that REM-related neural activity is associated with later developmental outcomes^9,10^. In animal studies, pontine (PGO-like) waves and hippocampal theta oscillations during REM sleep have been demonstrated to exhibit phase-locking, suggesting that eye movement-related activity reflects coordinated neural rhythms characteristic of REM sleep^18^. For example, premature neonates with lower REM activity have significantly lower mental development index scores at 6 months of age^9^. In addition, an electroencephalography (EEG) study in term-born infants reported that REM sleep–related EEG characteristics measured at 4 months of age predicted language and social communication at 18 months^19^. These observations suggest that REM-linked processes continuously support neurodevelopment from the prenatal period onward.

REM sleep in early life is crucial for synaptic development and neural plasticity, supporting the refinement of sensorimotor and subcortical circuits, such as the brainstem and hippocampus^20^. It is generated by brainstem nuclei, including the pedunculopontine tegmental nucleus and locus coeruleus^21,22^, which are essential for the development of subcortical networks, such as the basal ganglia and limbic system—regions implicated in the expression of RRB^23,24^. Fetal eye movement activity during active sleep—considered a precursor of REM sleep—may influence the early organization of brainstem projections to these subcortical structures and their cortical targets, supporting the development of self-regulation and behavioral flexibility^21,22,25^. The observed negative association between fetal EMD and RRBs aligns with prior imaging studies showing altered basal ganglia morphology in children with high RRB scores^23^. Recent evidence further indicates that altered functional connectivity in prefrontal–limbic circuits, including prefrontal–amygdala connectivity, is linked to executive function deficits that mediate the severity of RRBs in ASD^26^. Disruption of the broader regulatory circuit, including the anterior cingulate cortex (ACC) and prefrontal circuits, may impair executive attention and inhibitory control, thereby contributing to the emergence and severity of RRBs^27,28^.

Early sleep organization has been linked to language development, with sleep–wake consolidation during infancy supporting receptive and expressive language acquisition^29,30^. The receptive and expressive language KIDS domains reflect vocabulary comprehension and production, sentence use, and communicative behaviors relevant to everyday interactions. The receptive subscale primarily assesses the understanding of spoken words and following verbal instructions, whereas the expressive subscale evaluates vocabulary use, sentence formation, and verbal expression of needs or emotions. Thus, these domains capture lexical and communicative language development aspects, providing important context for interpreting our findings^15,16^. Although previous studies have demonstrated that sleep supports phonological learning^30^, recent longitudinal research has further demonstrated that shorter nighttime sleep duration is associated with poorer cognitive and language development in early childhood^31^. Moreover, sleep-related neural activity across REM and NREM states is implicated in abstraction and language learning in infants, thereby providing a mechanistic framework beyond REM-specific effects^32^. Together, these findings suggest that adequate total sleep and REM-related neural activity contribute to the consolidation of emotional and cognitive memory networks essential for language acquisition^24^. Therefore, the association between fetal EMD and language ability may reflect the contribution of prenatal REM activity to the maturation of cognitive control networks. These include the brainstem, limbic system, ACC, and prefrontal circuits involved in attention, emotion, and executive functions^27,28^. Consistent with recent theoretical work, sleep during early development appears to prioritize neural reorganization with a transition toward repair and clearance functions around 2–3 years of age^33^, which may help to contextualize why early sleep organization relates to later language outcomes. REM sleep may indirectly support the maturation of neural structures essential for language acquisition, and fetal EMD could serve as a promising biomarker for this developmental process.

Additionally, EMD was associated with infant sleep characteristics at 1 year of age, particularly delayed sleep onset time, indicating a potential trend toward continuity from fetal REM to postnatal sleep regulation. Previous studies have highlighted associations between sleep disturbances and developmental problems, including ASD^34^. Although these studies emphasize postnatal sleep problems as risk factors, our findings suggest that reduced fetal REM activity may precede sleep dysregulation, indicating a potential prenatal origin of impaired sleep–wake regulation and its downstream developmental consequences. A study on mice reported that the neurons involved in REM/NREM sleep and wakefulness originate from a common developmental lineage in the hindbrain^35^. This may indicate that sleep–wake regulatory circuits begin to differentiate prenatally and form an integrated basis for later sleep-state transitions, including sleep onset. This developmental link underscores the value of fetal EMD as a potential marker for sleep-regulatory maturation. In this study, a significant association between fetal EMD and sleep outcome was observed at 1 year of age but not at 6 months or at 3 years. The pattern observed here differs from our previous findings at 1.5 years, where lower fetal EMD was associated with more frequent night awakenings rather than later sleep onset^36^. These differences may reflect variation in sleep indicators or developmental stages assessed, as nighttime awakenings tend to decline after infancy while bedtime timing becomes more stable. Additionally, differences in questionnaire format and environmental factors, such as childcare attendance, may have contributed to this inconsistency.

These results complement prior theoretical frameworks that emphasize the interpretive value of fetal behavior as an index of neurodevelopmental health^37^. Notably, in our previous study^11^, we analyzed separate participant cohorts recruited during different study periods with no overlap in individuals. Despite this complete independence of the population and differences in the age of developmental assessment (1.5 years vs. 3 years), similar associations were observed between fetal EMD and subsequent developmental challenges. This consistency across fully independent samples underscores the potential robustness and generalizability of fetal EMD as a biomarker for early neurodevelopmental risk.

Some limitations should be acknowledged, including a small sample size and reliance on caregiver-reported neurodevelopmental assessments.

An important limitation is that we inferred fetal behavioral state based solely on eye movement patterns without electrophysiological confirmation. Following established developmental sleep science nomenclature^4^, the states we measured represent active behavioral states—precursors to mature REM sleep—rather than definitive sleep stages, which cannot be reliably identified until 3–6 months postpartum. Additionally, we did not differentiate between slow and rapid eye movements in our EMD calculation. Although rapid eye movements are considered more specific markers of active/REM states, our inclusive measure encompasses heterogeneous eye movement types with potentially different neurophysiological origins^12^. Future studies should include velocity thresholds to determine whether REM-specific activity shows stronger predictive validity than overall eye movement density. Nevertheless, despite these methodological limitations, EMD has demonstrated meaningful associations with neurodevelopmental outcomes in both our current study and previous research^3,9,11^, suggesting that it may capture developmentally relevant neural activity patterns even as a composite measure.

In addition, our cohort was drawn from a single clinical center, limiting the extent of multivariate analysis and the generalizability of the results. Among the 67 participants with valid fetal EMD measurements, only 41 (61%) provided complete follow-up data at 3 years, mainly because of missing questionnaire responses rather than systematic exclusions. Although no significant demographic differences were identified between the follow-up and attrition groups (Supplementary Table S1), attrition may have introduced selection bias if families with greater developmental concerns or fewer resources were less likely to complete follow-up. Furthermore, because no standardized definition of infant sleep problems exists, our sleep indicators were defined based on prior research; moreover, breastfeeding data were unavailable, which may have influenced night awakening frequencies.Finally, because fetal EMD was derived from a single 60-min session, across-day reliability could not be assessed and should be examined in future studies. Future research with larger, multi-site cohorts and more objective longitudinal assessments is needed to validate and extend these findings.

In conclusion, this study is the first to report a longitudinal association between lower fetal EMD and both behavioral rigidity and language delays at 3 years of age. These findings support the emerging perspective that sleep-related neurophysiological processes beginning before birth contribute to subsequent developmental outcomes. Notably, our findings suggest that REM activity during the fetal period may predict the formation of neural networks involved in behavioral regulation and language development, indicating that fetal EMD has potential utility as an early screening biomarker for neurodevelopmental risk.

## Methods

### Ethical declaration

This study was conducted in accordance with the principles of the Declaration of Helsinki and approved by the Ethics Committee of Kyushu University Hospital (No. 21344). Written informed consent was obtained from all participants.

### Study participants

Pregnant women at more than 24 weeks’ gestation were recruited between October 2018 and October 2023. The inclusion criteria were singleton pregnancies receiving perinatal care at our institution and the absence of chromosomal abnormalities.

### Data acquisition and calculation of fetal EMD

To examine fetal EMs, we conducted 60-min ultrasonographic sessions between 34 and 36 weeks of gestation^11^. The participants were placed in a supine position in a quiet room and were allowed to adjust their posture as needed. All sessions were performed between 13:00 and 16:00, at least 2 h after eating, to minimize diurnal variation in fetal eye-movement activity reported in term fetuses^38^. This standardized timing aimed to enhance the reliability of a single-session assessment. Fetal EMs were observed using transabdominal two-dimensional sonography (APLIO 500 TUS-A500; TOSHIBA, Japan) with a 3.5-MHz convex transducer (PVT375BT Probe) at a frame rate of ≥ 30 frames per second. The recordings were saved in MP4 format on an SD card. The effective observation time was defined as the total duration for which the fetal EMs could be reliably assessed. Cases with ≤ 80% effective observation time (≤ 48 min) were excluded because of insufficient data quality. Fetal EMs were counted, and time-series data were generated from the recorded videos. Each minute was analyzed to identify “EM periods,” defined as intervals containing at least one EM. EMD was calculated as the total number of EMs divided by the total duration of the EM periods (in minutes), as previously described^3,11^.

### Developmental assessment

When children reached 3 years of age or the corrected age for preterm births, parents were mailed questionnaires to assess developmental outcomes. The Japanese versions of the KIDS and SRS-2 were used for assessment.

The KIDS Type T correlates with the Wechsler Preschool and Primary Scale of Intelligence and the Ages and Stages Questionnaire, third edition^[15,16]^, demonstrating acceptable concurrent validity with internationally recognized developmental measures. We used the Type T version of the KIDS, which was developed for assessing children with developmental delays. In this study, only items corresponding to the 2–4-year age range were extracted. Seven domains were evaluated in the KIDS: physical-motor, manipulation, receptive language, expressive language, linguistic concepts, social relationships with children, and social relationships with adults. The developmental age was calculated for each domain.

The SRS-2 is a standardized caregiver-reported questionnaire for screening autism spectrum disorder that can be used from 2.5 years of age. It comprises five subscales: social awareness, social cognition, social communication, social motivation, and restricted interests and RRBs. Each item is rated on a 4-point Likert scale, and higher scores indicate greater severity of autistic traits. The SRS-2 has demonstrated good reliability and validity across cultures and provides a quantitative measure of social and behavioral difficulties in children. For the total and domain-specific scores, a T-score of ≥ 60 was used as the cutoff to define abnormality^[17]^.

### Sleep problem assessment

Sleep-related variables were assessed using caregiver-reported questionnaires at 6 months and 1 year of age, and sleep logs at 3 years of age. The questionnaires administered at 6 months and 1 year were modified from the Brief Infant Sleep Questionnaire^39^ to assess bedtime, nighttime sleep duration, and night awakenings. For the 3-year assessment, sleep logs were considered valid if entries were recorded for at least 10 of the 14 consecutive days. The logs were paper-based, and caregivers were instructed to record entries every other day, including bedtime, wake-up time, and times of nighttime awakenings. The number of nighttime awakenings was calculated from these records.

Three sleep-related indicators were defined based on our previous study^11^ on early childhood sleep and development.

1. Bedtime after 22:00.
2. Nighttime sleep duration of ≤9 h (between 20:00 and 8:00).
3. Frequently awake at night.

Night awakenings were defined as follows:

- ≥3 awakenings per night at least thrice per week at 6 months and 1 year.
- ≥2 awakenings per week at 3 years.

### Covariates

Maternal age at delivery, gestational age at birth, birth weight, small for gestational age status, parity (0 or ≥ 1), and infant sex were obtained from medical records. Parity was recorded at the time of recruitment during pregnancy. These variables were selected as covariates based on previous studies indicating their associations with developmental disorders^40,41^. Because of the limited sample size, two covariates, maternal age and birth weight, were selected for the primary multivariate analysis based on their strong correlations with infant outcomes.

### Analysis

All analyses were conducted using JMP^®^ Pro 18 (SAS Institute Inc., Cary, NC, USA). *p* < 0.05 was considered statistically significant. Descriptive statistics were expressed as means with standard deviations for continuous variables and as numbers with percentages for categorical variables.

- **Outcome 1: developmental problem**
  Linear regression was used to examine the relationship between EMD and developmental age for each of the KIDS domains and the total and domain-specific T-scores of the SRS-2. Univariate analysis was conducted, followed by multivariate analysis, adjusting for maternal age and birth weight at delivery as covariates. Logistic regression analysis was conducted to examine the association between EMD and the total and domain-specific SRS-2 T-scores, which were categorized into two groups based on the cutoff values. Each variable was analyzed using univariate logistic regression. ORs and 95% CIs were also determined.
- **Outcome 2: sleep problem**
  Univariate logistic regression was conducted to assess the association between fetal EMD and each sleep indicator at 6 months, 1 year, and 3 years. ORs and 95% CIs were also determined.

## Supplementary Information

Below is the link to the electronic supplementary material.

Supplementary Material 1

## Data Availability

The data that support the findings of this study are available from the corresponding author upon reasonable request.

## References

[1] de Vries, J. I. P. & Fong, B. F. Normal fetal motility: an overview. *Ultrasound Obstet. Gynecol.* **27**, 701–711 (2006). 16710877 10.1002/uog.2740

[2] Inoue, M. et al. Functional development of human eye movement in utero assessed quantitatively with real-time ultrasound. *Am. J. Obstet. Gynecol.* **155**, 170–174 (1986). 3524238 10.1016/0002-9378(86)90105-5

[3] Okawa, H. et al. Eye movement activity in normal human fetuses between 24 and 39 weeks of gestation. *PLOS One*. **12**, e0178722 (2017). 28700709 10.1371/journal.pone.0178722 PMC5507482

[4] Kahn, A., Dan, B., Groswasser, J., Franco, P. & Sottiaux, M. Normal sleep architecture in infants and children. *J. Clin. Neurophysiol.* **13**, 184–197 (1996). 8714339 10.1097/00004691-199605000-00002

[5] Horimoto, N., Hepper, P. G., Shahidullah, S. & Koyanagi, T. Fetal eye movements. *Ultrasound Obstet. Gynecol.* **3**, 362–369 (1993). 12797264 10.1046/j.1469-0705.1993.03050362.x

[6] Yamazaki, R. et al. Evolutionary origin of distinct NREM and REM sleep. *Front. Psychol.* **11**, 567618 (2020). 33381062 10.3389/fpsyg.2020.567618 PMC7767968

[7] Ornitz, E. M. et al. The EEG and rapid eye movements during REM sleep in normal and autistic children. *Electroencephalogr. Clin. Neurophysiol.* **26**, 167–175 (1969). 4183370 10.1016/0013-4694(69)90207-7

[8] Tanguay, P. E., Ornitz, E. M., Forsythe, A. B. & Ritvo, E. R. Rapid eye movement (REM) activity in normal and autistic children during REM sleep. *J. Autism Child. Schizophr*. **6**, 275–288 (1976). 186448 10.1007/BF01543468

[9] Arditi-Babchuk, H., Feldman, R. & Eidelman, A. I. Rapid eye movement (REM) in premature neonates and developmental outcome at 6 months. *Infant Behav. Dev.* **32**, 27–32 (2009). 18996599 10.1016/j.infbeh.2008.09.001

[10] Chen, H. L. et al. Rapid eye movement sleep during early life: A comprehensive narrative review. *Int. J. Environ. Res. Public. Health*. **19**, 13101 (2022). 36293678 10.3390/ijerph192013101 PMC9602694

[11] Nakahara, K. et al. Association of fetal eye movement density with sleeping and developmental problems in 1.5-year-old infants. *Sci. Rep.* **12**, 8236 (2022). 35581284 10.1038/s41598-022-12330-1 PMC9114104

[12] Horimoto, N., Koyanagi, T., Satoh, S., Yoshizato, T. & Nakano, H. Fetal eye movement assessed with real-time ultrasonography: are there rapid and slow eye movements? *Am. J. Obstet. Gynecol.* **163**, 1480–1484 (1990). 2240091 10.1016/0002-9378(90)90609-b

[13] Charman, T. & Baird, G. Practitioner review: diagnosis of autism spectrum disorder in 2- and 3-year-old children. *J. Child. Psychol. Psychiatry*. **43**, 289–305 (2002). 11944873 10.1111/1469-7610.00022

[14] Zwaigenbaum, L. et al. Early intervention for children with autism spectrum disorder under 3 years of age: recommendations for practice and research. *Pediatrics* **136** (Suppl 1), S60–S81 (2015). 26430170 10.1542/peds.2014-3667E PMC9923898

[15] Miyake, K. A new test developmental screening scale -Kinder infant development scale-. *Hum. Dev. Res.* **6**, 147–163 (1990).

[16] Hashimoto, K. Validity of the family-rated kinder infant development scale (KIDS) for children. *Pediat Therapeut* 3 (2013).

[17] Bruni, T. P. Test review: social responsiveness scale–second edition (SRS-2). *J. Psychoeduc Assess.* **32**, 365–369 (2014).

[18] Karashima, A., Katayama, N. & Nakao, M. Phase-locking of spontaneous and tone-elicited Pontine waves to hippocampal theta waves during REM sleep in rats. *Brain Res.* **1182**, 73–81 (2007). 17919463 10.1016/j.brainres.2007.08.060

[19] Ventura, S. et al. Infant sleep EEG features at 4 months as biomarkers of neurodevelopment at 18 months. *Pediatr. Res.* **98**, 1474–1485 (2025). 39979586 10.1038/s41390-025-03893-6 PMC12549339

[20] Del Rio-Bermudez, C., Kim, J., Sokoloff, G. & Blumberg, M. S. Theta oscillations during active sleep synchronize the developing rubro-hippocampal sensorimotor network. *Curr. Biol.* **27**, 1413–1424e4 (2017). 28479324 10.1016/j.cub.2017.03.077 PMC5446093

[21] Segawa, M. Development of the nigrostriatal dopamine neuron and the pathways in the basal ganglia. *Brain Dev.* **22** (Suppl 1), S1–S4 (2000). 10984655 10.1016/s0387-7604(00)00149-2

[22] Peirano, P., Algarín, C. & Uauy, R. Sleep-wake States and their regulatory mechanisms throughout early human development. *J. Pediatr.* **143** (Suppl), S70–S79 (2003). 14597916 10.1067/s0022-3476(03)00404-9

[23] Calderoni, S., Bellani, M., Hardan, A. Y., Muratori, F. & Brambilla, P. Basal ganglia and restricted and repetitive behaviours in autism spectrum disorders: current status and future perspectives. *Epidemiol. Psychiatr Sci.* **23**, 235–238 (2014). 24816251 10.1017/S2045796014000171 PMC6998382

[24] Peirano, P. D. & Algarín, C. R. Sleep in brain development. *Biol. Res.* **40**, 471–478 (2007). 18575679

[25] Ninomiya, Y., Kayama, Y. & Koyama, Y. Postnatal development of cholinergic neurons in the mesopontine tegmentum revealed by histochemistry. *Int. J. Dev. Neurosci.* **23**, 711–721 (2005). 16289640 10.1016/j.ijdevneu.2005.09.002

[26] Zheng, X., Wang, X., Song, R., Tian, J. & Yang, L. Executive function, limbic circuit dynamics and repetitive and restricted behaviors in children with autism spectrum disorder. *Front. Neurosci.* **18**, 1508077 (2024). 39881807 10.3389/fnins.2024.1508077 PMC11774959

[27] Diamond, A. Developmental time course in human infants and infant monkeys, and the neural bases of, inhibitory control in reaching. *Ann. N Y Acad. Sci.* **608**, 637–669 (1990). discussion 669–676. 2075965 10.1111/j.1749-6632.1990.tb48913.x

[28] Fan, J., McCandliss, B. D., Sommer, T. & Raz, A. Posner, M. I. Testing the efficiency and independence of attentional networks. *J. Cogn. Neurosci.* **14**, 340–347 (2002). 11970796 10.1162/089892902317361886

[29] Dionne, G. et al. Associations between sleep-wake consolidation and Language development in early childhood: A longitudinal twin study. *Sleep* **34**, 987–995 (2011). 21804661 10.5665/SLEEP.1148 PMC3138173

[30] Knowland, V. C. P. et al. Sleep promotes phonological learning in children across Language and autism spectra. *J. Speech Lang. Hear. Res.* **62**, 4235–4255 (2019). 31770054 10.1044/2019_JSLHR-S-19-0098

[31] Smithson, L. et al. Shorter sleep duration is associated with reduced cognitive development at two years of age. *Sleep. Med.* **48**, 131–139 (2018). 29906629 10.1016/j.sleep.2018.04.005

[32] Mason, G. M., Lokhandwala, S., Riggins, T. & Spencer, R. M. C. Sleep and human cognitive development. *Sleep. Med. Rev.* **57**, 101472 (2021). 33827030 10.1016/j.smrv.2021.101472 PMC8164994

[33] Cao, J., Herman, A. B., West, G. B., Poe, G. & Savage, V. M. Unraveling why we sleep: quantitative analysis reveals abrupt transition from neural reorganization to repair in early development. *Sci. Adv.* **6**, eaba0398 (2020). 32948580 10.1126/sciadv.aba0398 PMC7500925

[34] Kikuchi, K. et al. Infants’ early recovery from sleep disturbance is associated with a lower risk of developmental delay in the Japan environment and children’s study. *Sci. Rep.* **14**, 17773 (2024). 39090186 10.1038/s41598-024-68672-5 PMC11294529

[35] Hayashi, Y. et al. Cells of a common developmental origin regulate REM/non-REM sleep and wakefulness in mice. *Science* **350**, 957–961 (2015). 26494173 10.1126/science.aad1023

[36] Henderson, J. M. T., France, K. G. & Blampied, N. M. The consolidation of infants’ nocturnal sleep across the first year of life. *Sleep. Med. Rev.* **15**, 211–220 (2011). 21051245 10.1016/j.smrv.2010.08.003

[37] diPietro, J. A. The psychophysiology of the maternal–fetal relationship. *Monogr. Soc. Res. Child. Dev.* **80**, 1–151 (2015).

[38] Morokuma, S., Horimoto, N., Satoh, S. & Nakano, H. Diurnal variation of eye movement and heart rate variability in the human fetus at term. *Early Hum. Dev.* **63**, 123–130 (2001). 11408101 10.1016/s0378-3782(01)00154-2

[39] Sadeh, A. A brief screening questionnaire for infant sleep problems: validation and findings for an internet sample. *Pediatrics* **113**, e570–e577 (2004). 15173539 10.1542/peds.113.6.e570

[40] Gardener, H., Spiegelman, D. & Buka, S. L. Perinatal and neonatal risk factors for autism: A comprehensive meta-analysis. *Pediatrics* **128**, 344–355 (2011). 21746727 10.1542/peds.2010-1036 PMC3387855

[41] Hua, J. et al. The prenatal, perinatal and neonatal risk factors for children’s developmental coordination disorder: A population study in Mainland China. *Res. Dev. Disabil.* **35**, 619–625 (2014). 24480608 10.1016/j.ridd.2014.01.001

