# Supplementary material for: Association between fetal eye movement density and developmental problems at age 3 years
Source: Sci Rep. 2026 Jan 16;16:5588. doi: 10.1038/s41598-026-35780-3 (PMC12890963; doi:10.1038/s41598-026-35780-3)
Supplement: Supplementary file 1 — Supplementary Material 1 [file 41598_2026_35780_MOESM1_ESM.pdf]

Association between fetal eye movement density and developmental problems at age 3 years

Yukiyo Shimada, Seiichi Morokuma, Kazushige Nakahara, Akiko Okuno, Kiyoko Kato

Supplementary Table S1. Comparison of baseline maternal, ultrasonographic, and birth characteristics between the follow-up (F) and dropout (D) groups.

Variable	Follow-up (F) Mean	SD*	Dropout (D) Mean	SD*	Difference (F−D)	p-value
**Maternal characteristics**						
Maternal age (years)	36.3	0.95	34.6	1.6	1.67	0.380
Parity (0 (n) / ≥1 (n))	26 (63.4%) / 15 (36.6%)		6 (42.9%) / 8 (57.1%)			0.410
**Ultrasonographic measurements**						
Gestational week at measurement	35.0	0.11	35.3	0.2	-0.33	0.120
Effective observation time (min)	57.0	0.45	56.1	0.8	0.95	0.295
EMD (n/min)	10.3	0.57	9.7	1.0	0.61	0.594
Eye movement time (min)	34.7	1.36	30.5	2.3	4.21	0.124
**Birth information**						
Sex of infant (Male / Female)	16 (39.0%) / 25 (61.0%)		5 (35.7%) / 9 (64.3%)			1.000
Birth weight (g)	2945.3	55.8	2983.1	95.5	-37.80	0.734
Type of delivery (Vaginal / Cesarean)	23 (56.1%) / 18 (43.9%)		8 (57.1%) / 6 (42.9%)			1.000
Apgar score at 1 min	8.0	0.13	8.0	0.2	0.02	0.925
Apgar score at 5 min	9.0	0.072	8.8	0.1	0.24	0.101
Umbilical cord pH	7.2990	0.0080	7.2960	0.0140	0.00	0.868

SD: standard deviation; EMD: eye movement density; min/minutes.

Data are presented as mean ± standard deviation (SD) or number (percentage). Comparisons between the follow-up (F) and dropout (D) groups were performed using Welch's t-test for continuous variables and Fisher's exact test for categorical variables. No significant differences were observed between the groups (all $p \geq 0.05$).
